# Feasibility Study of a Tablet-Based Intervention to Enhance the Meaning in Life Among Older Adults

**DOI:** 10.1093/geroni/igab046.984

**Published:** 2021-12-17

**Authors:** Seok In Nam, Sangyoon Han

**Affiliations:** Yonsei University, Seoul, Seoul-t'ukpyolsi, Republic of Korea

## Abstract

Recommendations to improve older adults' health and well-being focus on enhancing meaning in life through social interaction. Research studies have suggested that a tablet-based intervention can create opportunities to enhance meaning in life, thus reducing social isolation and loneliness. The purpose of this study was to examine the feasibility of using a tablet-based intervention to enhance meaning in life among older adults. Senior Meaning in Life Enhancement (SMiLE) is a tablet-based application developed and implemented based on person-centered counseling, logotherapy, and Acceptance Commitment Therapy (ACT). Thirty-one participants (adults aged over 65 years) were randomized for intervention (n = 15) or waitlist control (n = 16). The intervention group received a tablet with our embedded app. Participants were invited to participate in the app-based 2-month program for 30-minutes each day with the tablet. We evaluated pre-and post-semi-structured interviews, meaning in life scales, and usability tests. Data were analyzed using thematic analyses, descriptive statistics, and Mann–Whitney and Wilcoxon tests. Findings confirmed that at two months after the intervention, there was a statistically significant difference in the gap between pre-and post-meaning in life scores between the two groups (Z = -2.08, p < .05). Furthermore, qualitative findings included positive changes in behavior, relationships, and usability. This pilot study suggests the feasibility of a tablet-based intervention in older adults and demonstrates its potential benefit for meaning in life. These findings are valuable to researchers, practitioners, and designers interested in technological interventions for older adults.

